# Implementation of a proton FLASH platform for pre-clinical studies using a gantry-mounted synchrocyclotron

**DOI:** 10.1088/1361-6560/add106

**Published:** 2025-05-07

**Authors:** Arash Darafsheh, Anissa Bey

**Affiliations:** Department of Radiation Oncology, WashU Medicine, St. Louis, MO, 63110, United States of America

**Keywords:** proton therapy, FLASH, ultra-high dose rate, beam shaping

## Abstract

*Objective*. External beam radiation therapy (RT) at ultra-high dose rate (FLASH RT) has shown promise for improving the therapeutic ratio; exploiting its full potential, however, requires systematic preclinical studies to unravel the underlying radiobiological mechanisms. We demonstrate a proton irradiation platform for pre-clinical FLASH studies using a gantry-mounted proton therapy system in clinical operation. *Approach*. An accessory comprising a transmission ionization chamber, a tray accommodating beam modifying elements, including range shifting blocks made of boron carbide (B_4_C) and poly(methyl methacrylate) (PMMA), and brass apertures to shape the beam’s lateral extent was attached to the nozzle. A range modulator composed of arrays of holes drilled in a PMMA slab was used to form a spread-out Bragg peak (SOBP). The integral depth dose (IDD) curves, lateral dose profiles, and dose rate were measured using existing dosimeters for different beam modifying material combinations. *Results*. The range modulator allowed achieving an SOBP with 14 mm modulation. The proton range was gradually reduced through adding B_4_C and PMMA blocks in the beamline, while the beam spot’s size gradually increased and became more symmetric as protons traveled through more material. The commercial scintillator screen showed a dose-rate-independent response for measuring lateral dose profiles. The representative IDDs of the FLASH beam can be measured with a commercial multilayer ionization chamber device at a low dose rate since the IDD did not depend on the dose rate. *Significance*. This work demonstrated a platform for delivering ∼70 Gy s^−1^ SOBP proton FLASH beams using a gantry-mounted synchrocyclotron clinical system. We showed the evolution of an asymmetric and small single proton spot to a more symmetric and larger spot after ranging and shaping through different components. Using dosimeters commonly employed for quality assurance purposes, we report an efficient method for the characterization of proton FLASH beams.

## 1. Introduction

It is estimated that half of cancer patients would benefit from radiation therapy (RT) at some point during their treatment (Atun *et al* 2015). A crucial factor in RT is maintaining a delicate balance between eradicating the cancerous cells (tumor control probability) and radiation-induced toxicities to irradiated surrounding healthy tissues (normal tissue complication probability) (Baumann *et al* 2016). RT at ultra-high dose rate (UHDR), also known as FLASH RT delivering radiation at average dose rate ≳40 Gy s^−1^, has attracted intense research attention in the past decade due to the potential this treatment modality holds in improving the therapeutic ratio (Vozenin *et al* 2022, Farr *et al* 2022b, Di Martino *et al* 2024), i.e. reducing side effects while maintaining the same anti-tumor response, a phenomenon referred to as the ‘FLASH effect’. Safe and effective deployment of FLASH RT would significantly benefit from full understanding of its inherent radiobiology (Limoli and Vozenin 2023). Although the FLASH effect has been reported in multiple preclinical studies for various animal models and organs, its underlying mechanisms are yet to be fully elucidated (Favaudon *et al* 2022, Friedl *et al* 2022). Conducting such investigations, however, requires robust FLASH irradiation platforms and reliable associated dosimetry (Buchsbaum *et al* 2021, Fenwick *et al* 2024).

The feasibility of FLASH irradiation has been demonstrated using different radiation beam types, including electrons (Schüler *et al* 2022), photons (Rezaee *et al* 2021, Montay-Gruel *et al* 2022), protons (Diffenderfer *et al* 2022), and heavier ions (Tessonnier *et al* 2021, Weber *et al* 2022), through dedicated accelerators or modification of RT machines (Farr *et al* 2022a). Existing platforms for photon and electron FLASH irradiation enable studies for superficial targets. Proton therapy, thanks to Bragg peaks (BPs), has the dosimetric advantage of reducing the integral dose compared to state-of-the-art photon-based therapies (Faar *et al* 2018). In the context of FLASH RT, in the foreseeable future, using proton beams is more promising for treating deep-seated targets (Kim *et al* 2022). Currently, proton beams are used in ongoing prospective FLASH RT human clinical trials (Mascia *et al* 2023, Daugherty *et al* 2024).

Proton FLASH irradiation has been demonstrated using isochronous cyclotrons (Patriarca *et al* 2018, Yang *et al* 2023), synchrocyclotrons (Darafsheh *et al* 2020a, 2021), and synchrotrons (Yang *et al* 2022a). For proton UHDR delivery, two main approaches have been implemented: shoot-through (or transmission beam) (Verhaegen *et al* 2021, Kneepkens *et al* 2023) and ‘conformal’ FLASH RT (Schwarz *et al* 2022). The former places the target at the entrance or plateau region of the beam while the latter takes full advantage of the steep dose fall-off and locates the target in the spread-out BP (SOBP) region (Darafsheh *et al* 2021). In conventional proton therapy, treatments are eventually delivered through SOBPs, most commonly through the pencil beam scanning (PBS) technique. PBS, however, complicates the definition of the dose rate in proton FLASH RT (Deffet *et al* 2023). As such, for preclinical *in vivo* or *in vitro* studies involving small targets (∼1 cm^2^), using a static SOBP field (i.e. non-scanning) and a single spot is more straightforward (Hachadorian *et al* 2023). Typically, static fields can be created by modulating the range through passive scattering techniques, using stationary range modulation elements known as ‘ridge filters’ (Chu *et al* 1993).

Previously, we demonstrated feasibility of proton FLASH irradiation using a stationary superconducting synchrocyclotron in the manufacturer’s factory allowing a wide range of hardware modifications and tuning parameters in order to achieve UHDRs (Darafsheh *et al* 2020a, 2021). However, RT machines in clinical operation do not have the same degrees of freedom for FLASH irradiations. The purpose of this study is to demonstrate a robust platform for pre-clinical proton FLASH investigations using a gantry-mounted proton therapy synchrocyclotron in clinical operation. In particular, we demonstrate beam shaping of a full-energy pristine BP to produce an SOBP with a desirable range for pre-clinical studies through employing energy degrader blocks and a passive range modulator, made of an array of cavities in a poly(methyl methacrylate) (PMMA)slab. We demonstrate a reliable dosimetry technique using existing commercial dosimeters to characterize the FLASH radiation proton beam.

## 2. Materials and methods

### 2.1. Proton FLASH beam delivery system

**Figure 1.**
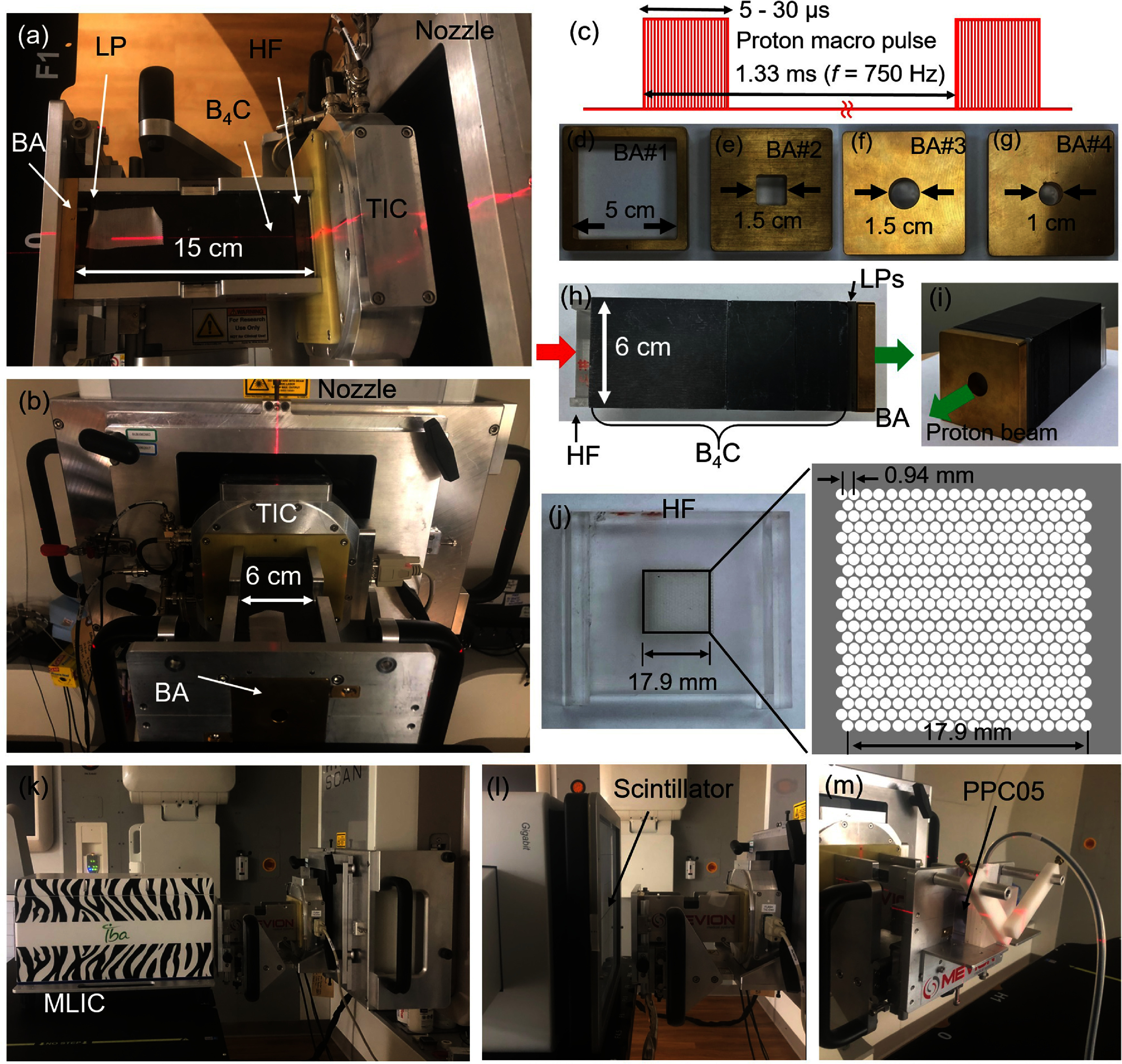
(a), (b) The FLASH accessory composed of a transmission ion chamber (TIC) at its proximal face to the nozzle, an aluminum tray housing beam shaping components: a range modulator (hole-filter, HF) used to form an SOBP, B_4_C and Lucite (LP) blocks (6 × 6 cm^2^ cross sectional area) to shift the proton beam range, and a brass aperture (BA) for beam shaping. (c) Macro pulse structure of the proton synchrocyclotron output radiation. (d)–(g) Brass apertures with 9.43 mm thickness and different opening shapes and sizes. (h), (i) An example arrangement of components in the proton beamline for configuration SOBP#8. The red and green arrows, respectively, show the proton beam path past the TIC and exit towards the irradiation target. (j) Range modulator hole-filter. (k) IDD measurement setup using the MLIC device, Zebra. (l) Lateral dose profile measurement setup using a scintillator screen coupled to a CCD camera device, Lynx. (m) Dose measurement setup using a parallel plate ionization chamber, PPC05.

A gantry-mounted superconducting synchrocyclotron (HyperScan, Mevion Medical Systems Inc., Littleton, MA, USA) in clinical operation was used as the irradiation platform (figures 1(a) and (b)). Several beam parameters were optimized by the vendor to enable FLASH mode delivery. Protons at a fixed energy of 227.1 MeV (32.2 cm range) are extracted from the accelerator into the nozzle. The temporal structure of the synchrocyclotron’s radiation output consisted of macro pulses with a nominal pulse repetition rate of 750 Hz corresponding to 1.33 ms interval between consecutive pulses, as schematically shown in figure 1(c). The charge-per-pulse can be adjusted through modifying the pulse duration, typically between 5–30 *μ*s, to set the average dose rate. Figures 1(a) and (b) show the ‘FLASH accessory’ mounted to the nozzle, which comprises a transmission ionization chamber (TIC). The FLASH accessory has an aluminum tray with a 6 cm × 6 cm internal cross sectional area to accommodate various passive components, such as the range modulator and energy degrader blocks with different thicknesses. Our measurements were performed with the gantry at 90°, however FLASH irradiation can be performed at other angles as well. In principle, in order to secure the components in the tray (mounted to the nozzle) against gravity, a robust cover can be designed.

### 2.2. Beam shaping accessories

Figures 1(d)–(j) show the various beam shaping components used in this work. The aluminum tray can accommodate up to 15 cm physical thickness of beam modifiers. A set of boron carbide (B_4_C) energy shifting blocks fabricated with specific thicknesses, 74.16, 37.08, 18.54, 9.27, and 4.63 mm, corresponding to 16, 8, 4, 2, and 1 mm water-equivalent thickness (WET), was used to reduce the proton range. The WET of B_4_C was determined to be approximately 0.216 times its physical thickness. To further reduce the beam residual range, a series of thin Lucite blocks each with 1 mm WET was used. To modulate the range and enable conformal FLASH dose distribution delivery through an SOBP beam, a ‘hole-filter’ (HF) was used. The HF is composed of an array of 400 holes with 0.8 mm diameter arranged over an area of 17.9 × 17.9 cm^2^ in 20 rows by 20 columns at the center of a PMMA slab with a 9.9 mm thickness as presented in figure 1(j). A spectrum of proton ranges is produced by the HF depending on the path length of the protons traversing the HF akin to that in the ‘ridge filters’. The thickness of the HF influences the SOBP width. Different brass apertures (BAs) with 9.43 mm physical thickness were used to shape the beam’s periphery: square shaped openings with 5 cm (BA#1) and 1.5 cm sizes (BA#2) (figures 1(d) and (e)), and circular shaped openings with 1.5 cm (BA#3) and 1 cm diameters (BA#4) (figures 1(f) and (g)).

In order to produce BP and SOBP proton FLASH beams with different residual ranges, various configurations of components, summarized in table 1, were placed in the tray. The corresponding SOBP# configurations consisted of the BP# configurations with the HF added at the proximal end of the tray to the nozzle to create the SOBP.

**Table 1.** Passive beam modifying material configurations stacked in the FLASH accessory’s tray. The range modulation hole-filter (HF) has a 9.9 mm PMMA thickness, and unless otherwise specified, was placed proximal to the nozzle’s exit. The beam range was measured at a low dose rate with the Zebra device in close contact with the BA#1 brass aperture. WET: water-equivalent thickness, BP: Bragg peak, SOPB: spread-out Bragg peak.

Configuration	Material block in the tray	WET (cm)	Range (cm)
BP#1	None	0	32.2
SOBP#1	HF	N/A	32.1
BP#2	18.7 mm B_4_C	4	28.18
SOBP#2	HF + 18.7 mm B_4_C	N/A	28.06
BP#3	37.4 mm B_4_C	8	24.21
SOBP#3	HF + 37.4 mm B_4_C	N/A	24.07
BP#4	74.34 mm B_4_C	16	16.46
SOBP#4	HF + 74.34 mm B_4_C	N/A	16.29
BP#5	111.74 mm B_4_C	24.1	8.60
SOBP#5	HF + 111.74 mm B_4_C	N/A	8.47
BP#6	130.44 mm B_4_C	28.2	4.69
SOBP#6	HF + 130.44 mm B_4_C	N/A	4.57
BP#7	139.84 mm B_4_C	30.2	2.64
SOBP#7	HF + 139.84 mm B_4_C	N/A	2.57
BP#8	139.84 mm B_4_C + 4 mm Lucite	34.2	2.22
SOBP#8	HF + 139.84 mm B_4_C + 4 mm Lucite	N/A	2.11
BP#9	139.84 mm B_4_C + 8 mm Lucite	38.2	1.80
SOBP#9	HF + 139.84 mm B_4_C + 8 mm Lucite	N/A	1.66

Since the FLASH accessory can accommodate a maximum of ∼15 cm thickness of beam modifiers, for combination SOBP#9, 4 mm (out of 8 mm) Lucite phantoms were placed immediately outside the tray after the BA. To study the impact of the HF’s position in the tray on the beam characteristics, in one configuration (#8) denoted SOBP#8*, the HF was placed at the distal end of the tray relative to the nozzle. Similarly, the effect of the BAs on the beam lateral profile and integral depth dose (IDD) distributions was investigated.

### 2.3. Beam characterization

Figures 1(k)–(m) represent the geometry of the experimental setup for the IDD, beam lateral dose profile, and dose rate measurement, respectively. A scintillator screen device (Lynx PT, IBA Dosimetry GmbH, Germany) was used to measure the beam lateral 2D profiles. Lynx consists of a gadolinium-based scintillating screen coupled to a charge-coupled device (CCD) camera, with an active detection area of 300 × 300 mm^2^ and a spatial resolution of 0.5 mm-per-pixel. The entrance window of Lynx, reposing on the treatment table, was centrally aligned to the FLASH accessory tray, and placed either immediately after the BA or at the isocenter to empirically assess the beam divergence. Single proton spots with 5, 10, 15, and 30 *μ*s pulse widths corresponding to average dose rates of approximately 1.4, 3.7, 13.7, and 66 Gy s^−1^ were delivered to probe the Lynx detector’s dose rate dependency. The purpose was to evaluate whether the Lynx device was suitable for FLASH measurements. The total dose at each delivery was set to avoid saturating the device. Lynx readout used the vendor software application (IBA myQA) with a dedicated plugin. Proton beam spot profiles were acquired using the ‘single shot’ measurement mode where Lynx acquires one image, resulting from signal integration over a specified frame duration. Frame durations were typically set to be quite short (<5 s) as commensurate with the prompt UHDR beam delivery. The detector’s camera aperture was set to 100%, that is to be fully open, as recommended by the vendor.

A multilayer ionization chamber (MLIC) device (Zebra, IBA Dosimetry GmbH, Germany) was used to measure the IDDs for the beam modifying material configurations considered in this study. Zebra comprises 180 air-vented plane-parallel ionization chambers spaced 2 mm apart, covering an effective proton range up to 33 cm in water. The distal range (R_90_) was defined at the 90% dose fall-off. The BP/SOBP data acquisition and analysis were carried out using the vendor’s OmniPro Incline software. The range measurements were performed at a low dose rate (LDR, 8 *μ*s pulse width, ∼2.8 Gy s^−1^ dose rate) since the Zebra device is not suitable for operation at UHDR due to significant ion recombination. However, it should be noted that under the same beam optics, the IDD is independent of the dose rate as it measures the total dose over the detector’s plane.

A parallel-plate, air-vented ionization chamber (PPC05, IBA dosimetry GmbH, Germany), featuring a 9.9 mm-diameter collection electrodes with 0.5 mm spacing between the electrodes and a 1 mm-thick entrance window was used to measure the proton FLASH dose and dose rate. The PPC05 was operated at a bias voltage of −400 V and read out by a Dose 1 electrometer (IBA dosimetry). The ionization chamber was inserted into a dedicated PMMA holder presenting 1 mm entrance, thus situating the effective point of dose measurement at 2 mm from the holder’s surface. A Lucite phantom of approximately 5 cm was also placed downstream the PPC05 (figure 1(m)). The average dose rate was calculated based on the measured delivered dose ($D$), number of pulses ($N$), and pulse repetition frequency ($f$) through ${\bar D_{{\text{avg}}}} = Df/N$. The instantaneous dose rate was calculated through ${\bar D_{{\text{ins}}}} = D/\left( {\tau N} \right) = {\bar D_{{\text{avg}}}}/\left( {\tau f} \right)$, where $\tau $ is the pulse width. Correction factors for temperature and pressure, polarity, and ion recombination were obtained as described previously (Darafsheh *et al* 2021). In particular, to estimate the polarity and ion recombination correction factors, measurements were performed according to the two-voltage method at −200 V and +400 V bias voltages, respectively.

Radiochromic films are reliable dosimeters for measurements at FLASH dose rates (Jaccard *et al* 2017, Darafsheh *et al* 2022, Romano *et al* 2022). Radiochromic film measurements were performed with Gafchromic™ EBT3 films (Ashland Inc., Bridgewater, NJ, USA). Films were placed immediately after the BA, perpendicular to the beam, with 4.5 cm PMMA phantoms placed beyond them. Film irradiations were performed at the FLASH dose rate with all four apertures. In order to verify the beam optics at various dose rates, film samples, sandwiched between phantoms, were also placed along the beam and irradiated sequentially at a low and FLASH dose rate. The exposed films were scanned in transmission mode at 300 dpi (0.084 mm per pixel) resolution as 48-bit Tagged Image File Format (TIFF) images using a flatbed scanner (Expression 10000XL, Epson Americana Inc., Long Beach, CA, USA) 48 h post-irradiation (León-Marroquín *et al* 2019a, 2019b, Darafsheh 2025). The optical density was obtained from the red color channel response. Since radiochromic films are known to have a linear energy transfer (LET) dependency for proton dosimetry (Darafsheh *et al* 2019, 2020b), calibration curves were established at 5 cm and 28 cm depths in phantom under clinical dose rates. Film analyses were undertaken using in-house-developed routines in Python (version 3.12.7).

## 3. Results and discussion

**Figure 2.**
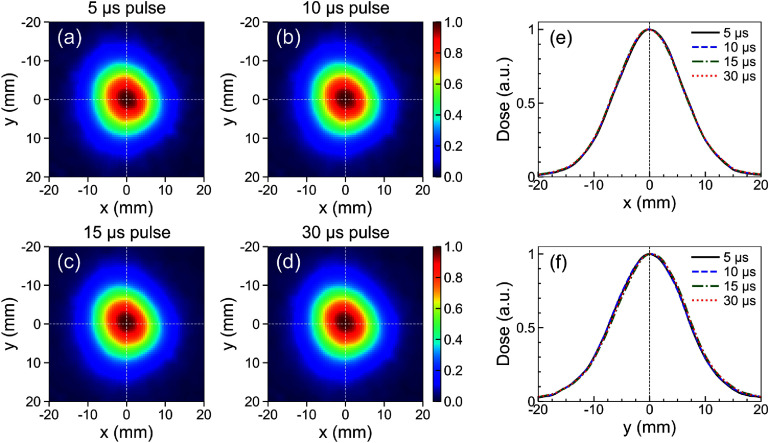
(a)–(d) Lateral 2D beam profiles and (e)–(f) corresponding 1D dose profiles, along the dashed lines, measured using the Lynx scintillator screen at close contact with the BA#1 aperture under different dose rates: (a) 5 *μ*s (∼1.4 Gy s^−1^), (b) 10 *μ*s (∼3.7 Gy s^−1^), (c) 15 *μ*s (∼13.7 Gy s^−1^), and (d) 30 *μ*s (∼66 Gy s^−1^) pulse widths. Measurements were performed for the SOBP#8 configuration (HF, 139.84 mm B_4_C, and 4 mm Lucite plates).

Figure 2 shows the FLASH proton beam lateral dose distribution measured by the Lynx scintillator screen placed at close contact with aperture BA#1 for SOBP#8 (*R*_90_ = 2.11 cm) at four different dose rates controlled by the radiation pulse width: 5, 10, 15, and 30 *μ*s. The selected pulse widths corresponded to 0.5, 1.3, 5.1, and 22.5 pC charge per pulse; which are equivalent to 0.38, 0.98, 3.8, and 16.9 mA beam currents at 750 Hz frequency. The respective average (instantaneous) dose rates were approximately 1.4 (373), 3.7 (493), 13.7 (1218), and 66 (2933) Gy s^−1^. No geometrical distortions were detected and the normalized spatial profiles along the *x*- and *y*-a*x*is directions were unaffected by the dose rate as shown in figure 2. This indicates that the Lynx device is suitable to operate under the FLASH dose rates considered in our work, for lateral beam profile (spot size or field size) characterizations, provided that the accumulated dose for each measurement is within the detector’s dynamic range (i.e. images are not saturated). The measured full-width at half-maximum (FWHM) along the crossplane (*x*) and inplane (*y)*-directions were on average 14.3 mm and 15.1 mm (at the exit of the aperture), respectively.

**Figure 3.**
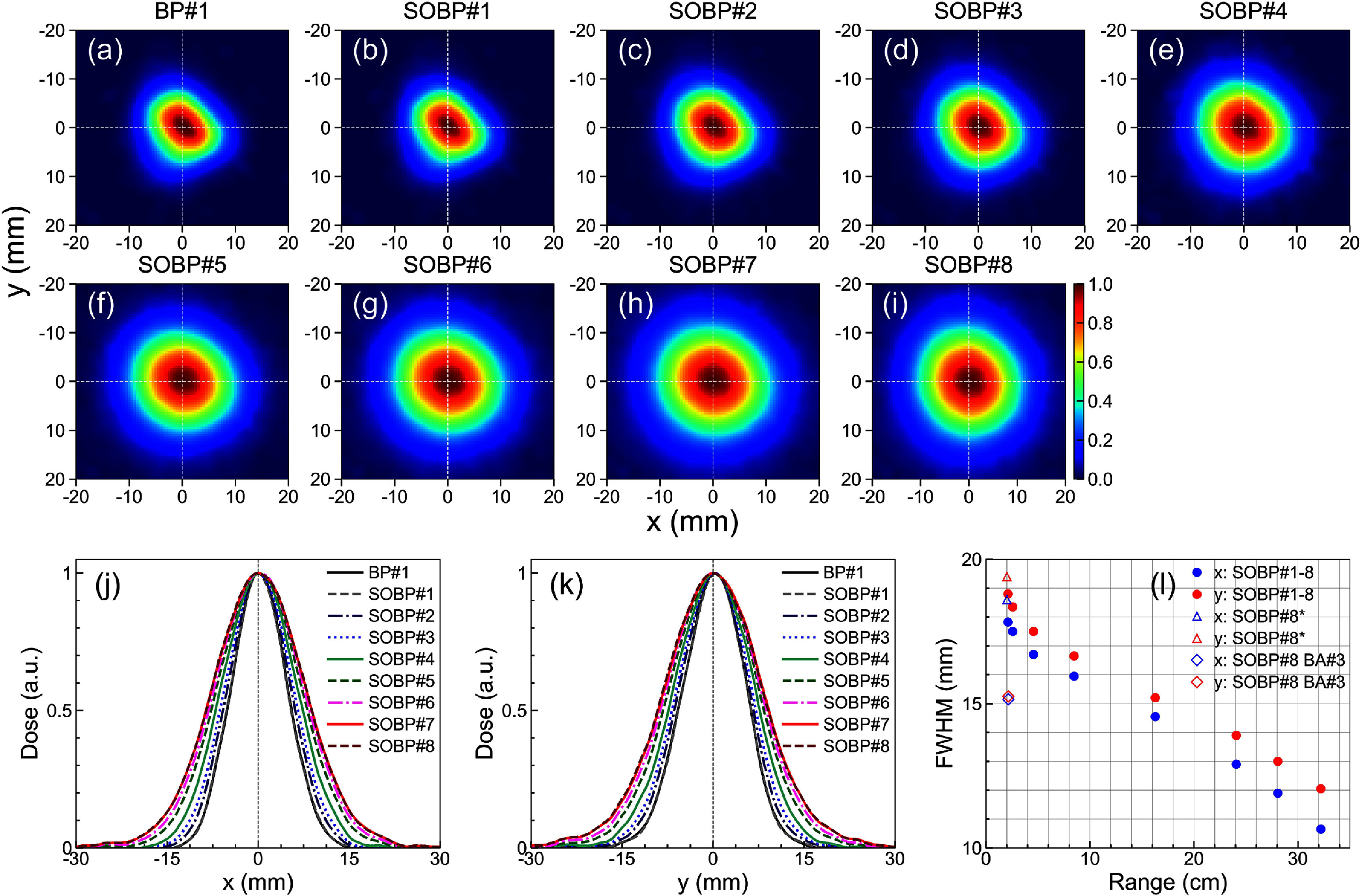
(a)–(i) 2D lateral dose (beam spot) distributions measured using the Lynx scintillator screen placed at the isocenter for different SOBP# material configurations in the beamline. (j), (k) 1D dose profiles along the *x*- and *y*- a*x*is, respectively. Characterizations were performed with the BA#1 aperture at the highest dose rate (30 *μ*s pulse width). (l) Spot size (FWHM) measured at the isocenter along the *x*- and *y*-a*x*is as a function of the range for the SOBP beams #1 through #8. For SOBP#8*, the hole filter (HF) was placed at the end of the FLASH tray (in all other cases the HF was at the beginning of the tray). One SOBP#8 set utilized the BA#3 aperture (in all other cases BA#1 was used).

Evolution of the proton FLASH beam spot’s lateral dose profiles measured with the scintillator screen for the different range shifting material SOBP combinations is shown in figures 3(a)–(k). The full-energy spot (BP#1) results in a somewhat ellipsoid profile with 10.6 mm and 12.1 mm FWHM at the isocenter along the *x*- and *y*-a*x*is, respectively. However, as more materials is placed in the beam path, the profile becomes more symmetric and larger due to multiple Coulomb scattering as shown in figures 3(j)–(k). For example, the SOBP#8 profile reaches 17.8 mm and 18.8 mm FWHM at the isocenter along the *x*- and *y*-directions, respectively.

The spot size as a function of the beam range is shown in figure 3(l) for different combinations of energy degraders in the beamline. The spot size increases gradually and linearly as the beam traverses more material thickness. When more phantoms are inserted in the tray, the spot becomes more symmetric due to multiple Coulomb scattering and the differences between the *x*-axis and *y*-axis spot size (FWHM) is gradually reduced. In order to confine the beam lateral spread, collimating BAs can be used. In that case, the resulting profile is defined by the aperture size, e.g. approximately 15 mm for BA#3 (figure 3(l)). The slight difference between the physical opening size of the BA and the measured spot size with Lynx stems from the beam divergence at the tray’s exit as measurements were performed at the isocenter.

**Figure 4.**
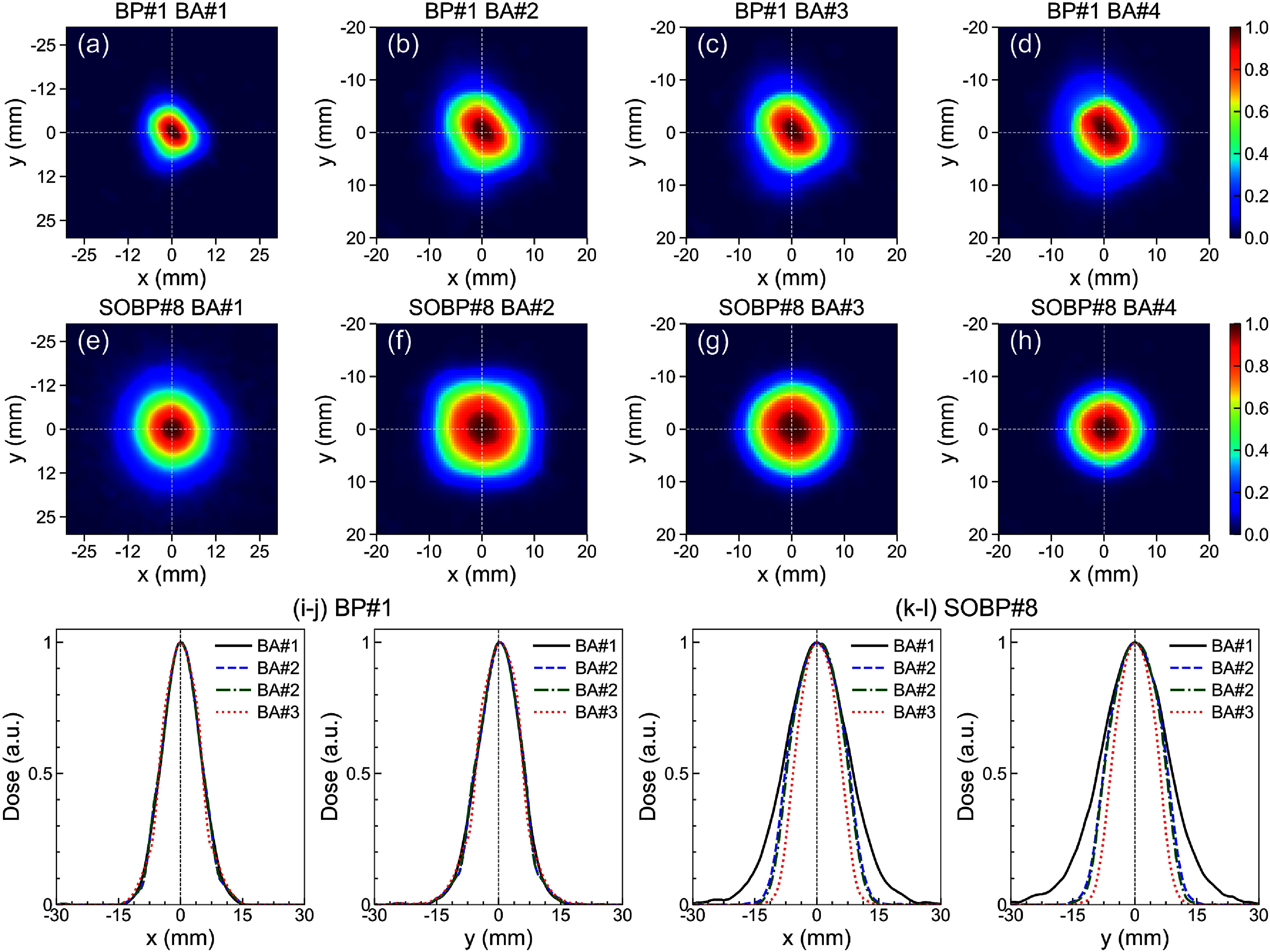
Lateral 2D dose distributions measured using the Lynx scintillator screen placed at the isocenter for (a)–(d) the full-energy BP#1 and (e)–(h) SOBP#8 configurations using different brass apertures (BA#1–4). (i)–(l) The corresponding 1D lateral dose profiles along the dashed white lines in each panel.

A series of BAs were used to shape the beam laterally. Figure 4 shows the effect of BAs on the beam lateral profile for material configurations BP#1 (empty tray) and SOBP#8. Since the beam spot is smaller than the BA#1’s opening, no visible impact was noted in figures 4(a)–(c), whereas in figure 4(d) using the 1 cm-diameter BA#4, a slight change in the dose map is noticeable. For the SOBP#8 configuration, the BAs’ beam shaping effect is readily significant as evidenced in figures 4(f)–(h) and (k)–(l). In principle, the BA collimators are suitable for shaping the FLASH proton beam for static small-field applications.

**Figure 5.**
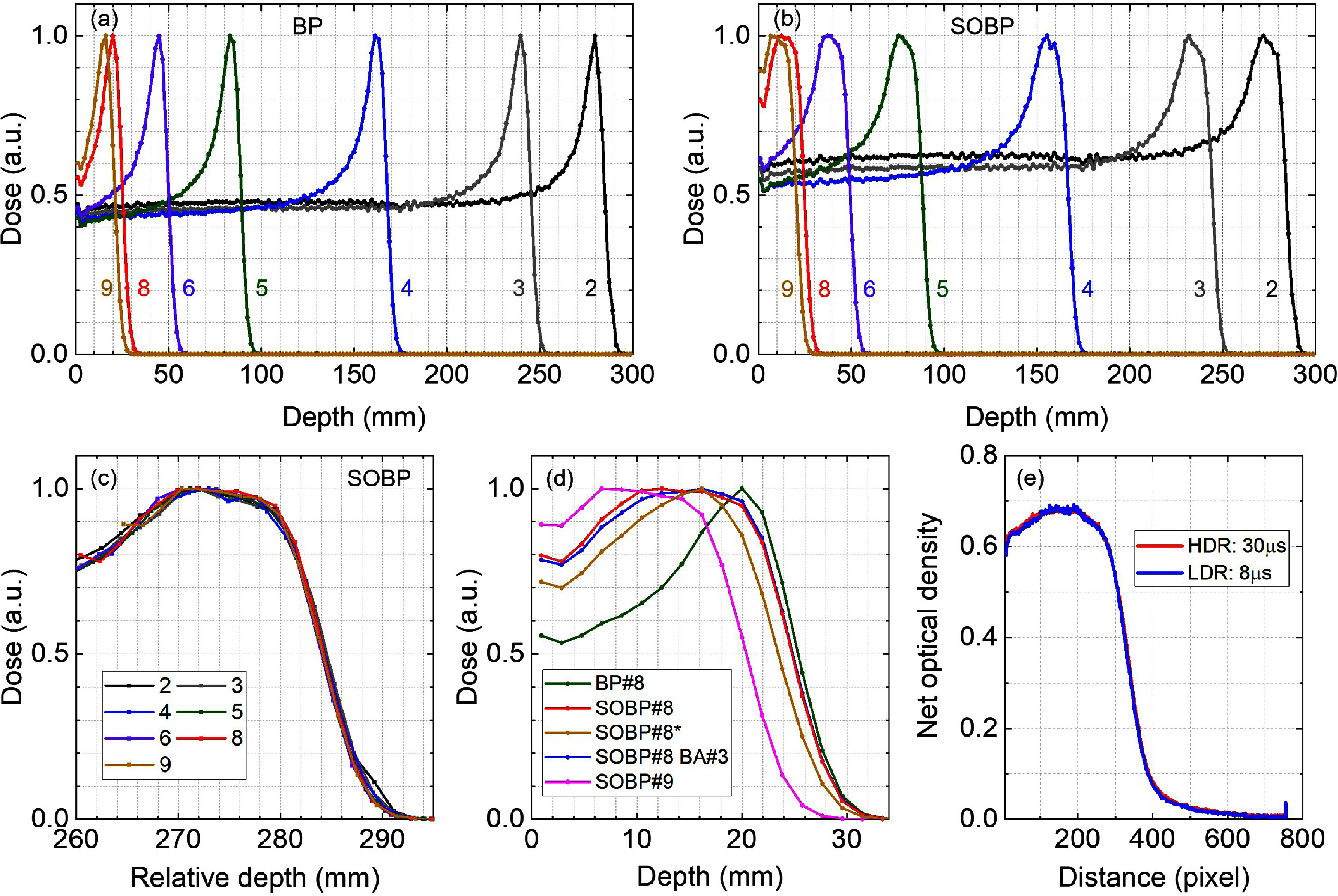
Integral depth-dose (IDD) curves measured with the Zebra device in close contact with the BA#1 aperture for different material combinations at clinical, low dose rate (LDR) 8 *μ*s pulse width (∼2.4 Gy s^−1^). (a) Pristine Bragg peak (BP) IDD profiles for 7 different WETs. (b) Spread-out Bragg peak (SOBP) IDD profiles corresponding to the BP configurations in (a) with the addition of range modulator hole filter (HF) at the beginning of the FLASH tray. (c) Expanded view of (b) in which dose profiles were shifted laterally to overlay (the depth values on the *x*-axis are relative). (d) IDD curves of the BP#8, SOBP#8 and SOBP#9 configurations. For ‘SOBP#8 +BA#3’, BA#3 was used to define the field size. For SOBP#8*, the HF was placed furthest from the nozzle in the tray. (e) Net optical density profiles measured for SOBP#8 configuration using the radiochromic dosimetry film at low dose rate (LDR) and high dose rate (HDR).

Figures 5(a)–(d) show the measured IDD profiles for different combination of materials placed in the FLASH accessory. The full energy pristine beam (BP#1) at the nozzle exit has a 32.2 cm range in water. However, since the MLIC device in our clinic has not been calibrated to measure ranges greater than 30 cm, the 18.7 mm B_4_C (4 cm WET) block was utilized to indirectly measure the full range. The range is reduced according to the WET of the materials in the tray. Figure 5(a) shows the pristine BP#, where the range modulating HF component was not inserted in the tray. In figure 5(b), the HF was placed in the tray immediately after the TIC (i.e. furthest upstream to the Zebra’s entrance window), as such the profiles exhibit an SOBP pattern. Figure 5(c) represents the expanded view of figure 5(b) over the last 3.5 cm of the IDD curves revealing SOBPs with 14 mm modulation (90%–90%). Due to more multiple Coulomb scatterings taking place, effectively smoothing the spectrum of energies, the SOBPs are formed. The impact of the BA on the IDD was negligible as shown in figure 5(d). On average, the range for the SOBP IDDs was ∼1 mm shorter than their BP counterparts, most likely due to additional energy loss introduced by the HF. Monte Carlo simulations would elucidate the nature of the observed difference; however, such study is beyond the scope of the present work.

The commercial MLIC device suffers from ion recombination losses and is therefore not suitable for FLASH dosimetry. It should be noted that prototypes of special MLICs designs have shown promise for IDD measurements under UHDR fields (Zhou *et al* 2022, 2024, Yang *et al* 2022b). The reported measurements were performed at a low dose rate (LDR, 8 *μ*s pulse width, ∼2.4 Gy s^−1^) to avoid the issues arising in UHDR, i.e. the non-negligible charge recombination. Nevertheless, the IDD is not expected to change with the dose rate, as it depends on the amount of material in the beamline and the proton range extracted from the cyclotron which has a fixed energy. We verified that the beam optics did not change with the dose rate using radiochromic film measurements as shown in figure 5(e).

In order to investigate the effect of the HF position in the tray, two extreme cases were studied considering SOBP#8. Measurements were performed with the HF placed at the proximal (SOBP#8) and distal (SOBP#8*) end of the tray to the nozzle, i.e. furthest upstream or downstream, respectively. The HF placement was found to not significantly impact the lateral dose profile, however, it appreciably affected the modulation depth as presented in figure 5(d) IDD curve SOBP#8*. The optimum position for the HF further upstream allows more range modulation providing a relatively flatter SOBP.

**Figure 6.**
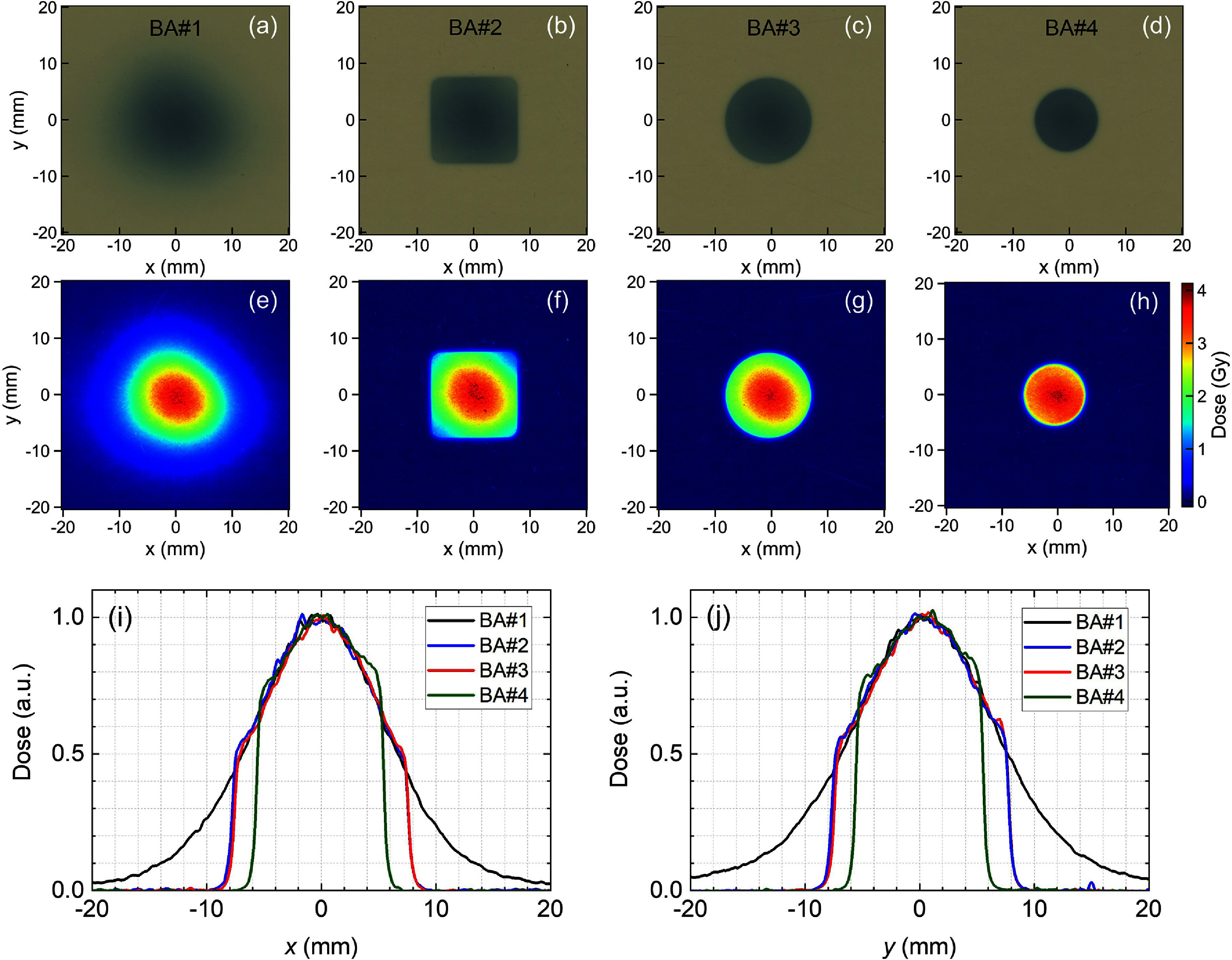
Lateral proton FLASH beam dose profiles measured using radiochromic film dosimeters placed immediately after the brass apertures with openings: (a), (e) 5 cm square, (b), (f) 1.5 cm square, (c), (g) 1.5 cm-diameter circle, and (d), (h) 1 cm-diameter circle. The corresponding central-axis 1D profiles along the (a) *x*-axis and (b) *y*-axis.

Figure 6 shows the proton FLASH beam lateral dose profile measured with different BAs for SOBP#8 using radiochromic films. The central axis dose was not significantly impacted by the BA. The lateral profile, however, shows a sharp fall-off due to the BA shaping effect. The film profiles are sharper than those obtained with the Lynx device (figure 4) since the latter’s surface was located at the isocenter, while the film measurements were performed at contact with the BA. Furthermore, the Lynx’x effective spatial resolution is 0.5 mm-per-pixel, while the 300-dpi-scanned EBT3 films resulted in a 0.084 mm-per-pixel resolution.

**Figure 7.**
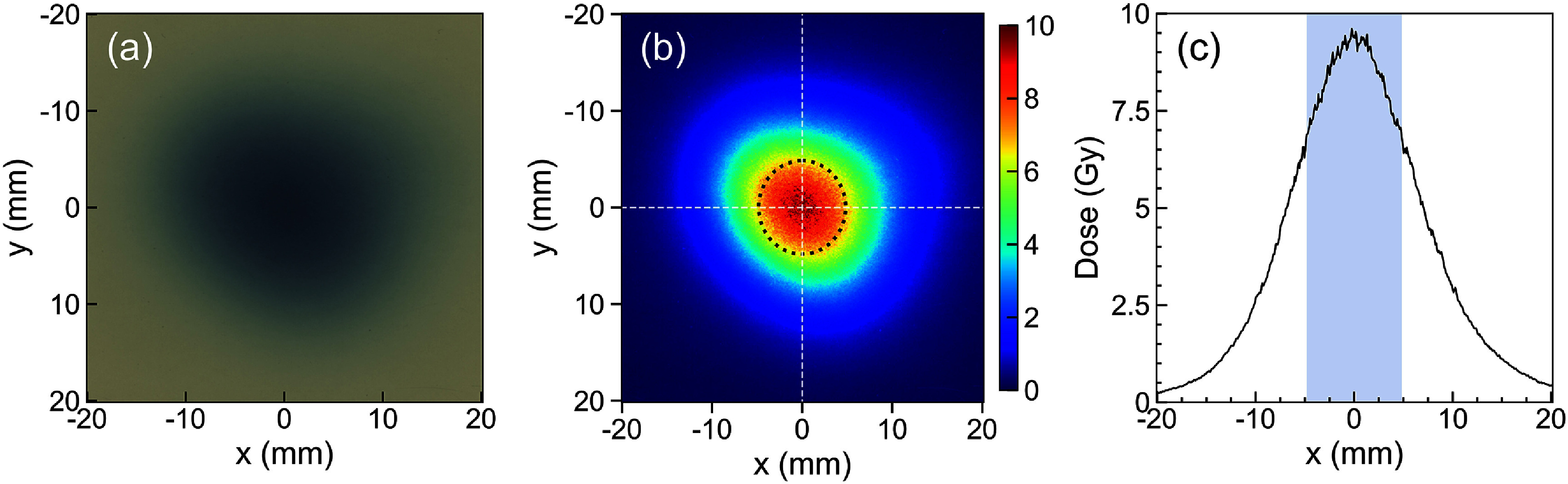
(a)–(b) Lateral 2D dose profile measured with the radiochromic film, where the PPC05 ionization chamber active area diameter is overlaid as the dashed-line circle and (c) the 1D dose profile along the *x*-axis. (The shaded area indicates the PPC05 active area).

In order to measure the dose rate, the PPC05 (figure 1(m)) was used with ∼5 cm Lucite phantoms placed behind the chamber’s PMMA holder. The proton FLASH beam was delivered for the SOBP#8 configuration. A Lucite phantom of 1-cm thickness was placed in front of the PPC05 chamber to measure the dose rate at the middle of the SOBP. The dosimetry protocol has been described in our previous work (Darafsheh *et al* 2021). The measured average dose rate in the SOBP region was 66 Gy s^−1^, which at 30 *μ*s pulse width, corresponds to 2933 Gy s^−1^ instantaneous dose rate. Due to the small beam spot size and its spatially inhomogeneous profile, we used radiochromic film to investigate the impact of volume averaging in the ionization chamber. Figure 7 shows the results of the film dosimetry. The average dose over pixels within the 9.9 mm-diameter central area corresponding to the PPC05 collecting electrode diameter was calculated, based on which the estimated average dose rate was 64 Gy s^−1^. However, the average dose rate around the proximity of the central axis of the beam is approximately 75 Gy s^−1^, a manifestation of volume averaging effect in the ionization chamber. Further work is ongoing to flatten the beam profile over a 1 cm target area.

Below is a summary and our recommendations for proton FLASH dosimetry using the existing commercial dosimeters investigated in this work.

**Radiochromic film:** Although it has been demonstrated that radiochromic films can be used for UHDR dosimetry (Jaccard *et al* 2017, Darafsheh *et al* 2022, Romano *et al* 2022), their dose-rate-independence should be evaluated for the beam type and dose rates of interest. This can be accomplished by delivering the same dose to the films at different dose rates and evaluating either the optical density or calibration curves. A Faraday cup can be placed behind the films to measure the beam current and to verify that the same dose (related to the total charge) has been effectively delivered to the films.

**Scintillator screen:** Dosimeters based on scintillation screens, such as the Lynx PT device, are commonly used in proton therapy for beam lateral dose profile measurements (Darafsheh *et al* 2024). Our results, obtained for proton FLASH irradiation produced by a synchrocyclotron, showed that as long as the acquired images are not saturated (i.e. the delivered dose is within the detector’s dynamic range), the Lynx device can be used to measure the lateral profiles for FLASH beams. The dose-rate-independence of Lynx should be verified for the beam type, radiation pulse structure, and dose rates of interest.

**Multi-layer ionization chamber:** MLIC devices, such as Zebra, are commonly used to measure IDDs for conventional dose-rate proton therapy beams. Their performance at UHDR is adversely affected by significant ion recombination. However, as long as the IDD does not change with the dose rate (e.g. the same beam optics is used for conventional and FLASH beams), which can be verified through radiochromic film measurements, an MLIC device can be used to ‘indirectly’ measure the IDD of a FLASH beam. In that case, the IDD measured at a low dose rate (within the dynamic range of the device) can be used as a representative IDD of a FLASH dose rate for the same beam energy. It should be noted, however, that due to quenching effect in radiochromic films as a result of higher LET near the BP, films are not reliable for IDD measurements. Nevertheless, the purpose of film verifications in this step is to show that the overall IDD shape remains the same regardless of the dose rate.

**Parallel-plate ionization chamber:** At UHDRs associated with FLASH beams, charge recombination in ionization chambers needs to be carefully evaluated. In principle, ion recombination can be reduced by using chambers with smaller gaps between the collecting electrodes and/or increasing the bias voltage of the electrodes. For examples, the Advanced Markus chamber (PTW Freiburg GmbH, Germany) with 1 mm gap was found to have a higher ion recombination compared to the PPC05 with 0.5 mm gap (Darafsheh *et al* 2021). In general, the following procedure can be used to evaluate ion recombination for a given chamber irradiated with a high dose rate beam. The same dose should be delivered at a clinical dose rate LDR and at the desired FLASH dose rate, HDR (this can be verified by a Faraday cup). Since the dose is the same, the ion recombination at the FLASH dose rate can be evaluated by comparing the two measurements (Petersson *et al* 2017).

Finally, it should be noted that when working with small fields with spatially non-uniform dose profiles, volume averaging effects can occur in the ionization chamber leading to dose under-estimation. In such cases, film dosimetry or Monte Carlo simulations can help to shed more light on the required corrections to measure the dose at the center of the beam.

## 4. Conclusion

A FLASH proton irradiation platform for pre-clinical studies capable of delivering SOBP beams at ∼70 Gy s^−1^ dose rate over an approximately 1 cm^3^ target volume using a gantry-mounted synchrocyclotron was demonstrated. We presented a practical technique for beam shaping employing passive components, as well as FLASH beam characterization using existing dosimeters including: radiochromic films, a multi-layer ionization chamber, a scintillation screen, a parallel-plate ionization chamber, and a Faraday cup. Further platform optimization is ongoing to create a more uniform lateral dose distribution through introducing additional passive components in the beamline.

## Data Availability

The data that support the findings of this study are available upon reasonable request from the corresponding author.
